# Neonatal death: Case definition & guidelines for data collection, analysis, and presentation of immunization safety data

**DOI:** 10.1016/j.vaccine.2016.03.040

**Published:** 2016-12-01

**Authors:** Jayani Pathirana, Flor M. Muñoz, Victoria Abbing-Karahagopian, Niranjan Bhat, Tara Harris, Ambujam Kapoor, Daniel L. Keene, Alexandra Mangili, Michael A. Padula, Stephen L. Pande, Vitali Pool, Farshad Pourmalek, Frederick Varricchio, Sonali Kochhar, Clare L. Cutland, The Brighton Collaboration Neonatal Death Working Group

**Affiliations:** a Medical Research Council: Respiratory and Meningeal Pathogens Research Unit, Johannesburg, South Africa; b Department of Science and Technology, National Research Foundation, Vaccine Preventable Diseases, South Africa; c Faculty of Health Sciences, University of the Witwatersrand, Johannesburg, South Africa; d Departments of Pediatrics, Molecular Virology and Microbiology, Baylor College of Medicine, Houston, TX, USA; e Global Clinical Research & Development – Epidemiology Novartis/GSK Vaccines, Amsterdam, The Netherlands; f Program for Appropriate Technology in Health (PATH), Seattle, USA; g Immunization and Vaccine Preventable Diseases, Public Health Ontario, Canada; h Immunization Technical Support Unit – Ministry of Health and Family Welfare, Public Health Foundation of India, New Delhi, India; i Department of Pediatrics, Faculty of Medicine, University of Ottawa, Ontario, Canada; j Tufts University School of Medicine, Boston, MA, USA; k Division of Neonatology, The Children's Hospital of Philadelphia and University of Pennsylvania, USA; l Ministry of Health Uganda, Soroti Regional Referral Hospital, Uganda; m Sanofi Pasteur Inc., Swiftwater, PA, USA; n School of Population and Public Health, University of British Columbia, Vancouver, Canada; o Independent Consultant, Vaccinologist, USA; p Global Healthcare Consulting, New Delhi, India

**Keywords:** *Neonatal death*, Maternal immunization, Adverse event, Guidelines, Case definition

## Abstract

More than 40% of all deaths in children under 5 years of age occur during the neonatal period: the first month of life. Immunization of pregnant women has proven beneficial to both mother and infant by decreasing morbidity and mortality. With an increasing number of immunization trials being conducted in pregnant women, as well as roll-out of recommended vaccines to pregnant women, there is a need to clarify details of a neonatal death. This manuscript defines levels of certainty of a neonatal death, related to the viability of the neonate, who confirmed the death, and the timing of the death during the neonatal period and in relation to immunization of the mother.

## 1 Preamble

### 1.1 Need for developing case definitions and guidelines for data collection, analysis, and presentation for *neonatal death* as an adverse event following immunization

Classification of a child's life into well-defined periods has become an important standardization to determine the care and interventions necessary to increase the chances of child survival. The neonatal period, which is globally accepted as beginning at birth and ending at 28 completed days of life [1], is recognized as the most vulnerable time in an infant's life. *Neonatal death* has been defined by the World Health Organization (WHO) as “deaths among live births during the first 28 completed days of life” [1] which can be further sub-divided into early neonatal deaths (deaths between 0 and 7 completed days of birth) and late neonatal deaths (deaths after 7 days to 28 completed days of birth) [2].

Although global neonatal mortality rates have declined, from 31.9 (95% confidence interval [CI] 31.9–32.8) deaths per 1000 live births in 1990 to 18.4 (95% CI 17.6–19.2) deaths per 1000 live births in 2013 [3], this rate of decline (40%) lags the progress made in decreasing mortality in children aged 1–59 months (56%) [2]. The contribution of neonatal deaths (2.8 million in 2013) to the under-5 deaths has increased from 37.4% in 1990 to 41.6% in 2013 [2], [3]. This trend has been projected to continue with anticipated further rapid declines in under-5 mortality. The first week of life is the most critical for a neonate with 36% of neonatal deaths occurring (1 million) in the first 24 h of life, 37% (1 million) occurring between days 1 and 7 of life and 27% (0.8 million) occurring between day 7 and day 27 of life in 2013 [2], [4].

**Fig. 1:**
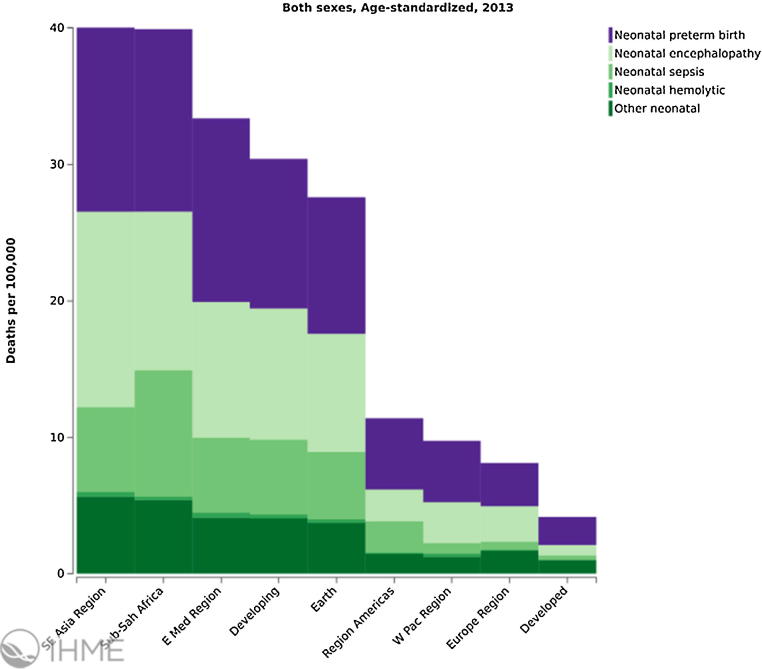
Neonatal mortality rate in 100,000, age-standardized, both sexes, World Health Organization regions, 2013/Institute for Health Metrics and Evaluation (IHME). GBD Compare. Seattle, WA: IHME, University of Washington, 2015. Available from http://ihmeuw.org/3qjx (accessed 21.12.15).

**Fig. 2:**
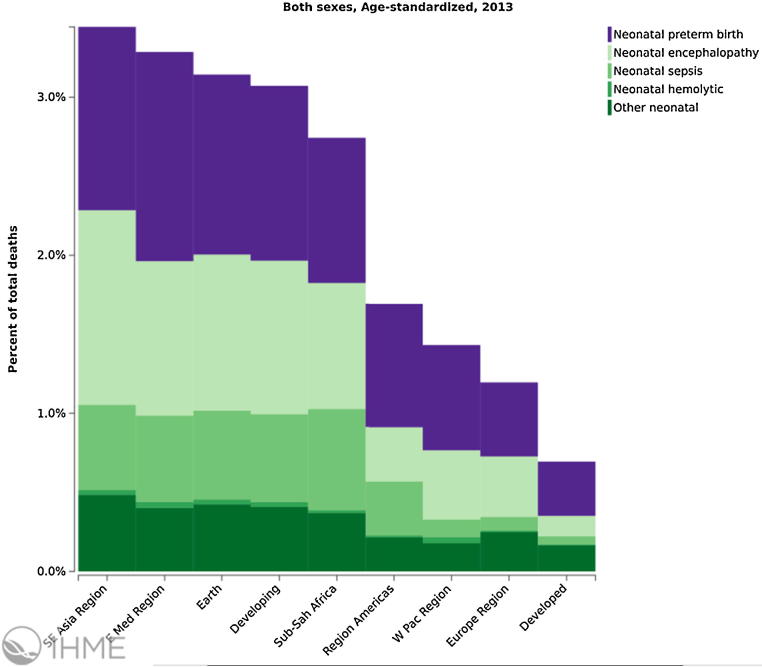
Neonatal mortality, percent of total deaths age-standardized, both sexes, World Health Organization regions, 2013/Institute for Health Metrics and Evaluation (IHME). GBD Compare. Seattle, WA: IHME, University of Washington, 2015. Available from http://ihmeuw.org/3qjy (accessed 21.12.15).

The leading causes of neonatal death in 2013 globally were (i) preterm birth complications (742,400), (ii) intrapartum related complications (neonatal encephalopathy from birth asphyxia/trauma, 643,800), and (iii) neonatal sepsis (346,400) and other neonatal infections [3] including pneumonia, tetanus and diarrhoea [2]. These global estimates mask the variations between countries and regions. For instance, only 7% of neonatal deaths in high-income countries are caused by infectious diseases, compared with 27% in Sub-Saharan Africa and 23% in Southern Asia [2] (Fig. 1, Fig. 2).

Immunization of pregnant women has been proven to be beneficial to the mother as well as the infant by decreasing morbidity and mortality during this vulnerable period [5], [6]. With the huge success of maternal tetanus vaccination for the prevention of neonatal tetanus [7], there has been significant resource allocation to vaccine research and development for pregnant women. Currently immunization of pregnant women with tetanus and influenza vaccines is widely recommended, with several countries also recommending pertussis vaccination [8], [9]. Other vaccines specifically targeting use during pregnancy are in various stages of development and include vaccines against Group B Streptococcus (GBS), Respiratory Syncytial Virus (RSV) and Cytomegalovirus (CMV).

The association between receipt of a vaccine during pregnancy and the subsequent death of the neonate requires documentation and investigation to assess if there are potential vaccine safety concerns which could have been associated with neonatal death. Adverse pregnancy outcomes, including neonatal death, can coincide temporally with immunization of pregnant women, and are therefore reported as adverse events in clinical trials. Clinical trials involving immunization of pregnant women have not, to date, shown any increase in incidence of neonatal deaths in infants of vaccine recipients compared to placebo recipients, or any increase above local incidence rate [10], [11], [12].

Despite a WHO definition of neonatal death being well accepted globally, an established, detailed definition for use in maternal immunization trials and surveillance following widespread implementation of maternal vaccinations does not currently exist. This is a missed opportunity, as data comparability across trials or surveillance systems would facilitate data interpretation and promote the scientific understanding of the event. The focus of vaccine safety monitoring in currently licensed vaccines has been on foetal death, pregnancy outcome (live birth or stillbirth), congenital anomalies and growth and development of the infant [12].

To determine association between *neonatal death* and maternal immunization, the background incidence of the adverse event needs to be established. *Neonatal death* reporting is a requirement for national and international statistical comparisons allowing countries to review their achievements in the area of maternal and neonatal health. It also allows measurement of effectiveness, or lack thereof, of interventions and practices. Although the WHO and the International Classification of Disease (ICD) have published definitions and recommendations for recording and reporting of live births and neonatal deaths, reporting criteria varies between countries. This is largely dependent on the understanding of the term at local/regional level, availability of resources, cultural and religious perceptions and practices [1], [12].

There are also differences in availability of trained health care workers and basic newborn care between high-income and low- and middle-income countries, which are not taken into account when global neonatal deaths are reported. Lack of clarity between the various terminologies used to assess pregnancy outcomes by health care workers leads to underreporting at several levels. Live birth, abortion, foetal death, stillbirth, perinatal death and neonatal death terms have definitions which may overlap depending on the setting. For instance, the WHO recommends registration of live births weighing at least 500 g or born at or after 22 weeks gestation [1]. However, in some cultures where neonatal survival is uncertain, the baby remains unnamed and unregistered, and if death occurs in the early neonatal period, will not be recorded or reported as a live birth. Similarly a live birth at less than 28 weeks of gestation in some countries will be reported as a miscarriage rather than as a live birth due to variation in national reporting criteria.

The case definitions for stillbirths, preterm delivery and neonatal infections in the context of maternal immunization are being defined by three other independent GAIA/Brighton Collaboration working groups (available at: http://www.brightoncollaboration.org).

Systematic review of the literature and stakeholder surveys of adverse events following immunization during pregnancy and the newborn period found a heterogeneity of outcome definitions, safety assessment methods, and data collection methods, as well as the lack of reporting consistency of safety data within and across studies was identified [13]. There is, therefore, a need to assess vaccine safety in the newborn of a mother who receives immunization during pregnancy; and a standardized definition of the adverse event, *neonatal death* following maternal immunization is required as well as data collection methods and tools. The WHO Initiative for Vaccine Research held a consultation to facilitate harmonization of key events for monitoring vaccine safety in pregnant women and newborn children in July 2014. In order to enable a standardized assessment and to improve comparability the *Neonatal Death Working Group* was established to define and develop guidelines to inform vaccine safety monitoring in clinical studies, post-licensure surveillance and in different geographical and cultural settings for the event neonatal death following maternal immunization during pregnancy.

### 1.2 Methods for the development of the case definition and guidelines for data collection, analysis, and presentation for neonatal death as an adverse event following maternal immunization

Following the process described in the overview paper [14] as well as on the Brighton Collaboration Website http://www.brightoncollaboration.org/internet/en/index/process.html, the Brighton Collaboration *Neonatal Death Working Group* was formed in 2015 and included 15 members of (clinical, academic, public health, industry) background. The composition of the working and reference group as well as results of the web-based survey completed by the reference group with subsequent discussions in the working group can be viewed at: http://www.brightoncollaboration.org/internet/en/index/working_groups.html.

To guide the decision-making for the case definition and guidelines, a narrative literature search without language restrictions was performed using Medline and Embase, employing the keywords: neonatal death, newborn death, perinatal death, infant death and maternal immunization and maternal vaccination. The search resulted in the identification of 972 Medline and Embase references following the removal of duplicates. Six hundred and ninety seven abstracts were screened for possible reports of *neonatal death* following maternal immunization. From these, 33 full text articles were reviewed in more detail, in order to identify studies using case definitions or, in their absence, providing clinical descriptions of the case material. This review resulted in a detailed summary of these articles, including information on the study type, the vaccine, the diagnostic criteria or case definition put forth for neonatal death.

Neonatal death as an adverse event following maternal immunization was not commonly monitored. Outcomes such as preterm birth, foetal death and stillbirth were the events that took precedent in most maternal vaccine clinical trials. In studies that did measure neonatal death, very few provided a referenced case definition.

### 1.3 Rationale for selected decisions about the case definition of neonatal death as an adverse event following immunization

#### 1.3.1 The neonatal period

Studies that did evaluate neonatal death, specified this event as *death of a live born infant before 28 completed days of age* [7], [15], [16], [17]. This well recognized neonatal period is adopted in the *neonatal death* case definition as this period has consistently been used in the literature reviewed, and in medical settings globally.

#### 1.3.2 The term *death*

The diagnosis of death or definition was not specified in any literature included. Death is regarded as the irreversible loss of the capacity for consciousness, combined with irreversible loss of the capacity to breathe including both cardiorespiratory death and brain death [18]. This definition of death can be applicable in a variety of economic settings which can be further expanded on if the situation requires it such as in a medicolegal context.

#### 1.3.3 The term neonatal death

Only two papers reviewed sub-classified neonatal deaths into early deaths defined as death of a newborn infant within the first week or first seven days of life [19], [20]. Late neonatal death was defined as death of a live born infant between 1 and 4 weeks of life [20].

We have further elaborated on this sub-classification. *Neonatal deaths* can be subdivided into (i) very early neonatal death (0 to <24 h), (ii) early neonatal deaths, occurring from the first day to the seventh day of life (≥24 h to <7 days), and (iii) late neonatal deaths, occurring after the seventh day but before 28 completed days of life (≥7 to <28 days). In the literature and in practice, neonatal death has only been classified into early (0–7 days) and late (>7 to 28 days) neonatal deaths [1], [21]. However in the context of maternal immunization and other factors associated with neonatal death (intrapartum events, prematurity and infections) a third classification of very early death can enable a more detailed analysis of adverse events surrounding a neonatal death.

#### 1.3.4 The term live births

The term “live birth” was infrequently mentioned in the literature as a prerequisite to classify a neonatal death. However, to correctly differentiate a neonatal death from a stillbirth and applicable to all geographic settings, the term live birth is necessary in the case definition, and an understanding and agreement of the term ‘live birth’ should be in place.

The Neonatal Death Working Group agreed to utilize the WHO and ICD-10 definition of live birth, which is: ‘the complete expulsion or extraction from its mother of a product of conception, irrespective of the duration of the pregnancy, which, after such separation, breathes or shows any other evidence of life – e.g. beating of the heart, pulsation of the umbilical cord or definite movement of voluntary muscles – whether or not the umbilical cord has been cut or the placenta is attached. Each product of such a birth is considered live born’ (http://www.who.int/healthinfo/statistics/indmaternalmortality/en/) [22].

#### 1.3.5 Timing of maternal immunization, birth and neonatal death

Three important time points need to be considered when describing the association between immunization of a pregnant woman and the adverse event, *Neonatal death* (i) date/time of immunization of the mother during pregnancy, which should be clearly recorded in antenatal records, (ii) date/time of birth of the infant and (iii) date/time (age) of infant at death.

Death within the first 24 h (<24 h) or first day of life should be recorded in units of completed minutes or hours of life. Deaths occurring ≥24 h of life should be recorded in days, from day 1 to 27 completed days (International Statistical Classification of Diseases and Related Health Problems-10th Revision, ICD-10) [22]. It is important to note that the first day of life is not equivalent to age 1 day. A newborn is only 1 day old if it has survived beyond 24 h of life. For example, if a newborn dies on the first day of life at 6 h after birth, age at death will be recorded as 6 h. If death occurs on the 27th day of life, the age at death will be 26 days. If the newborn dies on day 28 of life, age at death is 27 days.

A definition designed to be a suitable tool for describing relationships requires ascertainment of the outcome (e.g. n*eonatal death*) independent from the exposure (e.g. immunizations). Therefore, to avoid selection bias, a restrictive time interval from immunization to *Neonatal death* is not an integral part of such a definition. Instead, where feasible, details of this interval should be assessed and reported as described in the data collection guidelines.

Further, *neonatal death* often occurs outside the controlled setting of a clinical trial or hospital. In some settings it may be impossible to obtain a clear timeline of the event, particularly in less developed or rural settings. The Brighton Collaboration case definition avoids setting arbitrary time frames; however, the neonatal period is a pre-defined time period of the first 28 days of life. In order to avoid selecting against cases where exact number of days may not be available, less stringent measures of time have been included in level 3 definitions.

#### 1.3.6 Related term(s) of neonatal death

**Neonatal death** is a very specific and terminal event, however, the details available about the newborn and confirmation of the death may vary considerably between regions. This case definition is intended to accurately count the outcome *neonatal death* in various regions, and does not determine causality. Causality can often only be assessed at the time of data analysis, following collection of medical history, examination and interventional data prior and leading to the neonatal death. The case definition however includes details about the gestational age or weight, and confirmation of death as this information was regarded as necessary by the working group to accurately define a neonatal death.

**Neonatal death** is defined as the death of a live born infant, regardless of gestational age at birth, within the first 28 completed days of life. Each neonatal death can be further clarified into viable and non-viable deaths depending on the gestational age at which they were born, and where they were born.

**Medically-confirmed death**: death confirmed by suitable qualified medical or allied medical professional (including medical doctors, nurses, paramedics).

**Non-medically-confirmed death**: death confirmed by non-medically qualified person, including undertaker, community member, parent, family member, care-giver.

**APGAR scoring**: APGAR scoring of neonates in maternal immunization studies was rarely described in the literature [16], [17], [23]. Fell et al. [24] provided data on livebirths with 5-min APGAR below 7. Prone to high levels of subjectivity, a low score does not indicate death, as neonates with a 1 min score of 0 may score higher at the 5 min assessment. Due to this ambiguity, APGAR scores, though necessary to assess and document for appropriate management of the newborn, has not been utilized in the case definition or levels of diagnostic certainty as a pre-condition to classify a neonatal death.

**Gestational age**: Accurate gestational age measurement is a vital aspect in monitoring the progress of pregnancy and in decision making on management. The most common methods for determining gestational age were last (normal) menstrual period (LMP) or ultra sound scanning which was well described in the papers reviewed from well-resourced settings [15], [20], [25], [26]. Clinical examination of pregnant woman has also been used to determine gestational age [27]. Gestational age assessment remains a challenge in poorly resourced settings where LMP dates are unreliable and access to early ultrasound scanning and specialized health care workers is limited. Therefore recommendations on the methodology to determine gestational age applicable in various geographic and resourced settings have been described in the levels of diagnostic certainty (http://www.brightoncollaboration.org).

**Viability**: Reporting of neonatal deaths depends on reporting criteria in countries/regions. Identifying viability of a live born infant, as defined in the region/county, is an important precondition to classify a neonatal death to enable comparison between regions/countries.

Although the majority of papers reviewed did not clarify a viable gestation, the lowest gestations categorized as viable and potentially eligible for resuscitation and extensive medical intervention, were at either ≥20 weeks gestation [16], [17], [24] or ≥22 weeks gestation [19], [26], [27] from studies in well-resourced settings. Following death, this outcome will be reported as neonatal death. However, in countries with constraints of resources required to nurse extremely premature infants to a point where they could survive without medical intervention, live births at <28 weeks of gestational age or <1000 g birth weight are considered non-viable, and thus deaths of these neonates would be reported as abortions/miscarriages rather than neonatal deaths.

**Maternal immunization**: To report a neonatal death as an adverse event following maternal immunization, there needs to be evidence of vaccine administration during pregnancy. Evidence may, however, vary depending on the setting (e.g. clinical trial, post-marketing surveillance, routine antenatal vaccination). Thus, levels of diagnostic certainty of immunization during pregnancy have also been developed. Although pre-conceptual vaccination may have an impact on foetal or neonatal well-being, e.g. live (viral) vaccines, this report focuses on vaccines administered during pregnancy only.

#### 1.3.7 Formulating a case definition that reflects diagnostic certainty: weighing specificity versus sensitivity

It needs to be emphasized that the grading of definition levels is entirely about diagnostic certainty, not clinical severity of an event. Thus, a clinically very severe event, like *neonatal death*, may appropriately be classified as Level Two or Three rather than Level One if it could not reasonably confirm the details of the *neonatal death*.

As maximum specificity is normally associated with a loss of sensitivity, two additional diagnostic levels have been included in the definition, offering a stepwise increase of sensitivity from Level One down to Level Three, while retaining an acceptable level of specificity at all levels. The aim of this stepwise categorization is to optimize the capturing of neonatal deaths following maternal immunization.

### 1.4 Guidelines for data collection, analysis and presentation

As mentioned in the overview paper [14], the case definition is accompanied by guidelines which are structured according to the steps of conducting a clinical trial, i.e. data collection, analysis and presentation. Neither case definition nor guidelines are intended to guide or establish criteria for management of ill infants, children, or adults. Both were developed to improve data comparability.

The data collection guidelines are provided to enable an accurate classification and assessment of cause of neonatal death and in the process assess relatedness to maternal vaccination.

### 1.5 Periodic review

Similar to all Brighton Collaboration case definitions and guidelines, review of the definition with its guidelines is planned on a regular basis (i.e. every three to five years) or more often if needed.

## 2 Case definition of neonatal death [1] and immunization in pregnancy

For all levels of certainty, gestational age assessment should be based on Levels of certainty for gestational age which are being developed by the preterm delivery GAIA working group (refer to Preterm Birth case definition available at: http://www.brightoncollaboration.org).

### 2.1 Algorithm for classification of neonatal death

**Fig. 3:**
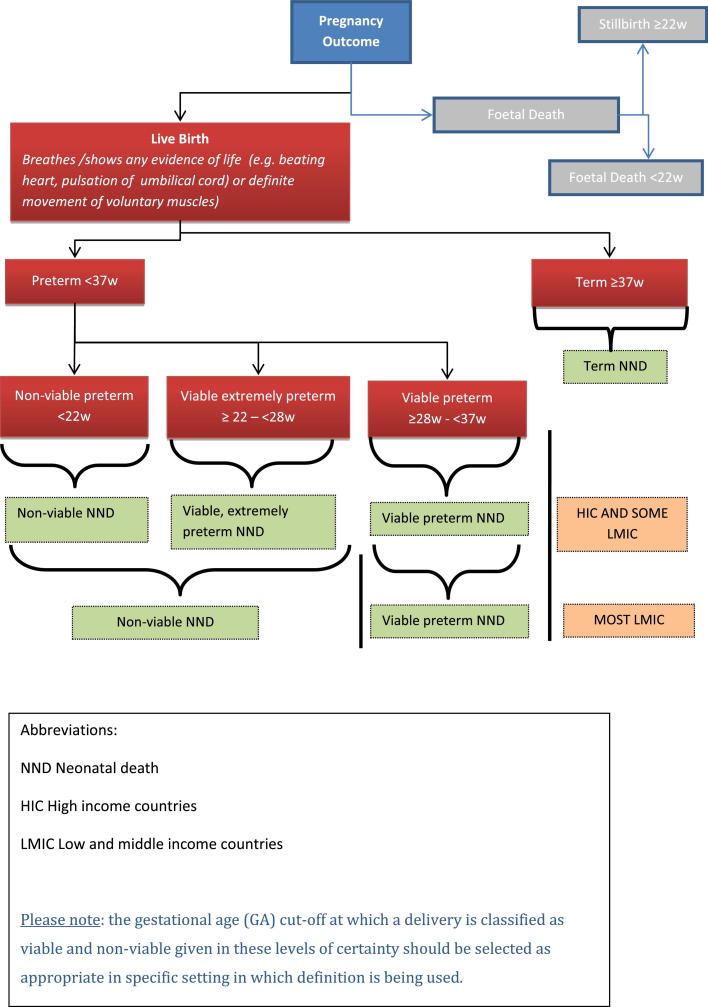
Algorithm for the classification of neonatal death.

Fig. 3 illustrates the algorithm for the classification of neonatal death.

### 2.2 Neonatal death in a non-viable live birth

**Level 1 of diagnostic certainty**

**Table:** 

1. Live born infant	AND
2. Gestational age <22 weeks (GA level of certainty = 1) [28]	OR
3. Birth weight <500 g	AND
4. Death of infant in first 28 days of life	AND
5. Medically-confirmed death	

**Level 2 of diagnostic certainty**

**Table:** 

1. Live born infant	AND
2. Gestational age/size of newborn assessed as at least one of:	
a. Gestational age <22 weeks (GA Level of Certainty = 1 OR 2) [28]	
b. Birth weight <500 g	AND
3. Death of infant in first 28 days of life	AND
4. Medically-confirmed death OR non-medically-confirmed death [3]	

**Level 3 of diagnostic certainty**

**Table:** 

1. Live born infant [1]	AND
2. Gestational age <5 months according to parent/family member/delivery attendant (GA Level of Certainty = 2 OR 3) [28]	AND
3. Death of infant in first 28 days of life	AND
4. Medically-confirmed death [2] OR non-medically-confirmed death	

### 2.3 Neonatal death in an extremely preterm live birth

**MAY apply to LMIC- or may be non-viable in LMIC** (Gestational age 22 to <28 weeks; should fit in with GA of stillbirth definition in region)

**Level 1 of diagnostic certainty**

**Table:** 

1. Live born infant	AND
2. Gestational age ≥22 and <28 weeks (GA Level of Certainty = 1) [28]	OR
3. Birth weight ≥500 g but <1000 g	AND
4. Death of infant in first 28 days of life	AND
5. Medically-confirmed death	

**Level 2 of diagnostic certainty**

**Table:** 

1. Live born infant	AND
2. Gestational age/size of newborn assesses as one or more of:	
a. Gestational age ≥22 and <28 weeks (GA Level of Certainty = 1 OR 2) [28]	
b. Birth weight ≥500 g but <1000 g	AND
3. Death of infant in first 28 days of life	AND
4. Medically-confirmed death OR non-medically-confirmed death	

**Level 3 of diagnostic certainty**

**Table:** 

1. Live born infant	AND
2. Gestational age ≥5 months but <7 months according to neonate's parent (mother/father)/family member/delivery attendant (GA Level of Certainty = 2 OR 3) [28]	AND
3. Death of infant in first 28 days of life	AND
4. Medically-confirmed death OR non-medically-confirmed death	

### 2.4 Neonatal death in a preterm live birth (gestational age ≥28 to <37 weeks)

**Level 1 of diagnostic certainty**

**Table:** 

1. Live born infant	AND
2. Gestational age ≥28 and <37 weeks (Level of Certainty = 1) [28]	OR
3. Birth weight ≥1000 g but <2500 g	AND
4. Death of infant in first 28 days of life	AND
5. Medically-confirmed death	

**Level 2 of diagnostic certainty**

**Table:** 

1. Live born infant	AND
2. Gestational age/size of newborn assesses as one or more of:	
a. Gestational age ≥28 and <37 weeks (GA Level of Certainty = 1 OR 2) [28]	
b. Birth weight ≥1000 g but <2500 g	AND
3. Death of infant in first 28 days of life	AND
4. Medically-confirmed death OR non-medically-confirmed death	

**Level 3 of diagnostic certainty** (MAY apply to LMIC- or may be non-viable in LMIC)

**Table:** 

1. Live born infant	AND
2. Gestational age ≥7 months but <9 months according to parent/family member/delivery attendant (GA Level of Certainty = 2 OR 3) [28]	AND
3. Death of infant in first 28 days of life	AND
4. Medically-confirmed death OR non-medically-confirmed death	

### 2.5 Neonatal death in a term live birth

**Level 1 of diagnostic certainty**

**Table:** 

1. Live born infant	AND
2. Gestational age ≥37 weeks (GA Level of Certainty = 1) [28]	AND
3. Birth weight >2500 g	OR
4. Documented intra-uterine growth retardation if ≤2500 g	AND
5. Death of infant in first 28 days of life	AND
6. Medically-confirmed death	

**Level 2 of diagnostic certainty**

**Table:** 

1. Live born infant	AND
2. Gestational age/size of newborn assesses as one or more of:	
a. Gestational age ≥37 weeks (GA Level of Certainty = 1 OR 2) [28]	
b. Birth weight ≥2500 g	AND
3. Death of infant in first 28 days of life	AND
4. Medically-confirmed death OR non-medically-confirmed death which is confirmed by examination by (by at least) non-medically-trained attendant (e.g. undertaker, community member)	

**Level 3 of diagnostic certainty (apply to LMIC)**

**Table:** 

1. Live born infant	AND
2. Gestational age ≥9 months according to parent/family member/delivery attendant (GA Level of Certainty = 2 OR 3) [28]	AND
3. Death of infant in first 28 days of life	AND
4. Medically-confirmed death OR non-medically-confirmed death	

### 2.6 Levels of certainty for diagnosis of maternal immunization

**Level 1 of diagnostic certainty**

**Table:** 

1. Woman confirmed as pregnant by positive pregnancy test or ultrasound confirmation	AND
2. Date/time of immunization of pregnant woman recorded in medical records by health care worker who administered/witnessed administration of vaccine	AND
3. Details of vaccine, including lot number, date of immunization	

**Level 2 of diagnostic certainty**

**Table:** 

1. Woman confirmed as pregnant by cessation of menstrual period and gravid uterus	AND
2. Date (at least month and year) of immunization of pregnant woman recorded in medical records by health care worker who administered/witnessed administration of vaccine	AND
3. Details of disease against which vaccinated	

**Level 3 of diagnostic certainty**

**Table:** 

1. Woman/medical attendant reports pregnancy	AND
2. Woman reports receipt of vaccination during pregnancy, but no formal recording of immunization available.	

## 3 Guidelines for data collection, analysis and presentation of neonatal death

It was the consensus of the Brighton Collaboration *Neonatal death Working Group* to recommend the following guidelines to enable meaningful and standardized collection, analysis, and presentation of information about Neonatal death. However, implementation of all guidelines might not be possible in all settings. The availability of information may vary depending upon resources, geographical region, and whether the source of information is a prospective clinical trial, a post-marketing surveillance or epidemiological study, or an individual report of Neonatal death. Also, as explained in more detail in the overview paper in this volume (available at: http://www.brightoncollaboration.org), these guidelines have been developed by this working group for guidance only, and are not to be considered a mandatory requirement for data collection, analysis, or presentation.

### 3.1 Data collection

These guidelines represent an ideal standard for the collection of data following immunization of pregnant women to allow for comparability of data, and are recommended as an addition to data collected for the specific study question and setting. It is acknowledged that not all data elements can be collected or may be necessary. The guidelines are not intended to guide the primary reporting of N*eonatal death* to a surveillance system or study monitor. Investigators developing a data collection tool based on these data collection guidelines also need to refer to the criteria in the case definition, which are not repeated in these guidelines.

Guidelines 1–38 below have been developed to address data elements for the collection of adverse event information as specified in general drug safety guidelines by the International Conference on Harmonization of Technical Requirements for Registration of Pharmaceuticals for Human Use [29], and the form for reporting of drug adverse events by the Council for International Organizations of Medical Sciences [30]. These data elements include an identifiable reporter and patient, one or more prior maternal immunizations, and a detailed description of the adverse event, in this case, of *Neonatal death* following immunization of the mother during pregnancy. The additional guidelines have been developed as guidance for the collection of additional information to allow for a more comprehensive understanding of *Neonatal death* following immunization of the mother during pregnancy.

Data on maternal participant (vaccinee/control) AND her infant(s) must be collected through maternal or family member interview, physical examination, review of medical and other available records as detailed further below.

#### 3.1.1 Source of information/reporter

For all cases and/or all study participants, as appropriate, the following information should be recorded:

1. Date of report.
2. Name and contact information of person reporting^2^ and/or diagnosing the *Neonatal death* as specified by country-specific data protection law.
3. Name and contact information of the investigator responsible for the participant, as applicable.
4. Relation to the maternal and neonatal participants/patient (e.g., immunizer [clinician, nurse], family member [indicate relationship], other).

#### 3.1.2 Vaccinee/control

##### 3.1.2.1 Demographics

For all cases and/or all study participants, as appropriate, the following information should be recorded:

5. Case/study participant identifiers of mother and newborn (e.g. first name initial followed by last name initial) or code (or in accordance with country-specific data protection laws).
6. For neonates: date of birth, date of death, age at death, sex, gestational age at birth, birth weight, if a twin/multiple gestation, birth order (1st twin, 2nd twin, 3rd triplet).

For mothers: Date of birth, date of death (in case of maternal death), age, parity, weight, mid-upper arm circumference (if required).

##### 3.1.2.2 Clinical and immunization history of mother and neonate

For all cases and/or all study participants (maternal and neonatal), as appropriate, the following information should be recorded:

7. Past medical history of both mother and neonate: including hospitalisations, underlying diseases/disorders, congenital anomalies, pre-immunization signs and symptoms including indicators for, or the absence of, a history of allergy to vaccines, vaccine components or medications; food allergy; allergic rhinitis; eczema; asthma (not exhaustive).
8. Past obstetric history: number of past pregnancies and outcome (live births, foetal deaths, abortions), type of gestation (singletons, multiple), pregnancy related illnesses and delivery complications, current status of past births (surviving, died (during neonatal period, post neonatal, under 5 death)).
9. Pregnancy details, including dates of last normal menstrual period, ultrasound examinations, gestation, antenatal care visits, pregnancy-related illnesses and complications (e.g. diabetes, hypertensive disorders, febrile illnesses, substance use/abuse, psychiatric conditions), type of gestation (singleton or multiple).
10. Labour and delivery details, including mode of delivery, evidence of infection (e.g. chorioamnionitis), complications (e.g. foetal distress, obstructed labour, antepartum/post-partum haemorrhage, assisted delivery) and outcome of delivery for both maternal and infant participants (including APGAR score (at 1 and 5 min), resuscitation requirement).
11. Any medication history (other than treatment for the event described) prior to, during, and after immunization including prescription and non-prescription medication as well as medication or treatment with long half-life or long term effect (e.g. immunoglobulins, blood transfusion and immunosuppressant).
12. Immunization history for maternal and newborn participants (i.e. previous immunizations and any adverse event following immunization (AEFI)), in particular occurrence of *Neonatal death* after a previous immunization during previous pregnancy.

#### 3.1.3 Details of the immunization

For all cases and/or all study participants (maternal and neonatal), as appropriate, the following information should be recorded by review of medical records or as reported by health care provider or patient:

13. Date and time of immunization(s).
14. Description of vaccine(s) (name of vaccine, delivery system, manufacturer, lot number, dose (e.g. 0.25 mL, 0.5 mL, etc.) and number of dose if part of a series of immunizations against the same disease), type and quantity of diluent used.
15. The anatomical sites (including left or right side) of all immunizations (e.g. vaccine A in proximal left lateral thigh, vaccine B in left deltoid).
16. Route and method of administration (e.g. intramuscular, intradermal, subcutaneous, and needle-free (including type and size), other injection devices).
17. Needle length and gauge.

#### 3.1.4 The adverse event neonatal death

18. For all cases at any level of diagnostic certainty and for reported events with insufficient evidence, the criteria fulfilled to meet the case definition should be recorded.

Specifically document:

19. Clinical description of signs and symptoms prior to *Neonatal death*, and if there was medical confirmation of the event(s) (i.e. neonate seen by physician).
20. Concurrent signs, symptoms, and diseases in the neonate.
21. Date/time of onset symptoms^3^ prior to neonatal death.
22. Date and time of death.^4^
23. Date and time of diagnosis of death (when death met case definition).^5^
24. Date and time of reporting of death.^4^
25. Measurement/testing of neonate done prior to death to assess causality.
  • Values and units of routinely measured parameters (e.g. temperature, blood pressure) – in particular those indicating the severity of the event.
  • Method of measurement (e.g. type of thermometer, oral or other route, duration of measurement, etc.).
  • Results of laboratory examinations, surgical and/or pathological findings, including autopsy findings and any diagnoses if present.
26. Any treatment (includes medication administered and procedures performed, e.g. intravenous antibiotics, mechanical ventilation, steroids) given to mother during pregnancy and to neonate prior to the *Neonatal death*, especially specifying the medication and dosing.
27. Exposures other than the immunization (either by the mother or the neonate if also immunized) 24 h before and after immunization (e.g. any medications, recreational drugs, food and environmental exposures) considered potentially relevant to the *Neonatal death*.
28. The event *Neonatal Death* [4] is always classified as “serious”.

#### 3.1.5 Miscellaneous/general

29. The duration of neonatal follow up until a *death* occurs should be predefined (e.g. for very early deaths monitor until 1 day old, for early neonatal death monitor until 7 days of age, for all neonatal deaths monitor until 28 days of age) based on:
  • Biologic characteristics of the vaccine e.g. live attenuated versus inactivated component vaccines; adjuvant versus non-adjuvant vaccines.
  • Biologic characteristics of the vaccine-targeted disease and effects on infected mothers and neonates.
  • Aetiology of *Neonatal death*(*s*) including patterns identified in previous trials (e.g. early-phase trials).
  • Biologic characteristics of the maternal vaccinee (e.g. nutrition, underlying disease like immunosuppressive illness).
30. Methods of data collection should be consistent within and between study groups, if applicable.
31. Investigators monitoring *Neonatal deaths* should provide guidance to reporters to optimize the quality and completeness of information provided.
32. Where possible, reports of *Neonatal death* should be collected throughout the neonatal period regardless of the time elapsed between maternal immunization and the adverse event. If this is not feasible due to the study design, the study periods during which safety data are being collected should be clearly defined.

## 4 Data analysis

The following guidelines represent a desirable standard for analysis of data on *Neonatal death* to allow for comparability of data, and are recommended as an addition to data analyzed for the specific study question and setting.

33. Data obtained from pregnant mothers receiving a vaccine should be compared with those obtained from an appropriately selected and documented control group(s) to assess background rates of neonatal death in non-exposed populations, and should be analyzed by study arm and dose where possible, e.g. in prospective clinical trials.
34. Reported events should be classified in one of the following five categories which includes the three levels of diagnostic depending on geographic setting and level of resources available. Events that meet the case definition should be classified according to the levels of diagnostic certainty as specified in the case definition. Events that do not meet the case definition should be classified in the additional categories for analysis.

**According to gestational age at birth of neonate event classification is in 4 categories**^6^

**A. Event meets case definition**: Neonatal death in a non-viable live birth

(1) Level 1: Criteria as specified in the Neonatal death case definition.
(2) Level 2: Criteria as specified in the Neonatal death case definition.
(3) Level 3: Criteria as specified in the Neonatal death case definition.

**Event does not meet case definition*****Additional categories for analysis***

(4) Reported *Neonatal death* with insufficient evidence to meet the case definition.^7^
(5) Not a case of *Neonatal death*.^8^

**B. Event meets case definition:** Neonatal death in an extremely preterm live birth: Gestational age 22 to <28 weeks (MAY apply to LMIC- or may be non-viable in LMIC)

(1) Level 1: Criteria as specified in the Neonatal death case definition.
(2) Level 2: Criteria as specified in the Neonatal death case definition.
(3) Level 3: Criteria as specified in the Neonatal death case definition.

**Event does not meet case definition*****Additional categories for analysis***

(4) Reported *Neonatal death* with insufficient evidence to meet the case definition.^7^
(5) Not a case of *Neonatal death*.^8^

**C. Event meets case definition**: Neonatal death in a preterm live birth (Gestational age ≥28 to <37 weeks)

(1) Level 1: Criteria as specified in the Neonatal death case definition.
(2) Level 2: Criteria as specified in the Neonatal death case definition.
(3) Level 3: Criteria as specified in the Neonatal death case definition.

**Event does not meet case definition*****Additional categories for analysis***

(4) Reported *Neonatal death* with insufficient evidence to meet the case definition [6].
(5) Not a case of *Neonatal death*.^8^

**D. Event meets case definition:** Neonatal death in a term live birth

(1) Level 1: Criteria as specified in the Neonatal death case definition.
(2) Level 2: Criteria as specified in the Neonatal death case definition.
(3) Level 3: Criteria as specified in the Neonatal death case definition.

**Event does not meet case definition*****Additional categories for analysis***

(4) Reported *Neonatal death* with insufficient evidence to meet the case definition.^7^
(5) Not a case of *Neonatal death*.^8^

35. The interval between immunization and reported *Neonatal death* could be defined as a composite of:
  35.1. The date/time of immunization to the date/time of birth AND.
  35.2. Date/time of birth to date/time of neonatal death.^4^

If few cases are reported, the concrete time course could be analyzed for each; for a large number of cases, data can be analyzed in the following increments:

**Subjects with Neonatal death: Interval between immunization and birth, and birth and Neonatal death (**Table 1**)**

36. The duration of the disease process prior to Neonatal death could be analyzed as the interval between the date/time of onset^2^ of the first symptoms and/or signs^3^ and the final outcome (neonatal death^4^ ^or^ 5) consistent with the case definition. Whatever start dates are used until the death, they should be used consistently within and across study groups.
37. If more than one measurement of a particular criterion is taken and recorded, the value corresponding to the greatest magnitude of the adverse experience could be used as the basis for analysis. Analysis may also include other characteristics like qualitative patterns of criteria defining the event.
38. The distribution of data (as numerator and denominator data) could be analyzed in predefined increments (e.g. measured values, times), where applicable. Increments specified above should be used. When only a small number of cases is presented, the respective values or time course can be presented individually.

### 4.1 Data presentation

These guidelines represent a desirable standard for the presentation and publication of data on Neonatal death following immunization of infant's mother to allow for comparability of data, and are recommended as an addition to data presented for the specific study question and setting. Additionally, it is recommended to refer to existing general guidelines for the presentation and publication of randomized controlled trials, systematic reviews, and meta-analyses of observational studies in epidemiology (e.g. statements of Consolidated Standards of Reporting Trials (CONSORT) [31], of Improving the quality of reports of meta-analyses of randomized controlled trials (QUORUM) [32], and of Meta-analysis Of Observational Studies in Epidemiology (MOOSE) [33], respectively.

39. All reported events of Neonatal death should be presented according to the categories listed in guideline 34.
40. Data on Neonatal death events should be presented in accordance with data collection guidelines 1–32 and data analysis guidelines 33–44.
41. Data should be presented with numerator and denominator (*n*/*N*) (and not only in percentages), if available.
  Although immunization safety surveillance systems denominator data are usually not readily available, attempts should be made to identify approximate denominators. The source of the denominator data should be reported and calculations of estimates be described (e.g. manufacturer data like total doses distributed, reporting through Ministry of Health, coverage/population based data, etc.).
42. The incidence of cases in the study population should be presented and clearly identified as such in the text.
43. If the distribution of data is skewed, median and inter-quartile range are usually the more appropriate statistical descriptors than a mean. However, the mean and standard deviation should also be provided.
44. Any publication of data on Neonatal death should include a detailed description of the methods used for data collection and analysis as possible. It is essential to specify:
  • The study design.
  • The method, frequency and duration of monitoring for Neonatal death.
  • The trial profile, indicating participant flow during a study including drop-outs and withdrawals to indicate the size and nature of the respective groups under investigation.
  • The type of surveillance (e.g. passive or active surveillance).
  • The characteristics of the surveillance system (e.g. population served, mode of report solicitation).
  • The search strategy in surveillance databases.
  • Comparison group(s), if used for analysis.
  • The instrument of data collection (e.g. standardized questionnaire, diary card, report form).
  • Whether the day of maternal immunization was considered “day one” or “day zero” in the analysis.
  • Whether the date the neonatal death occurred^4^ and/or the date of first observation of first symptoms leading to neonatal death^3^ used for analysis.
  • Use of this case definition for Neonatal death, in the abstract or methods section of a publication.^9^

## Figures and Tables

**Table 1:** Interval between immunization and birth, and birth and Neonatal death.

Interval between immunization and birth	Interval between birth and death	Number	Percentage
0–72 h	0–24 h		
	>24 h to 7 days		
	>7 to 28 days		
More than 72 h to 7 days	0–24 h		
	>24 h to 7 days		
	>7 to 28 days		
More than 7 days to 30 days	0–24 h		
	>24 h to 7 days		
	>7 to 28 days		
More than 30 days	0–24 h		
	>24 h to 7 days		
	>7 to 28 days		
Total			

## Footnotes

2 If the reporting centre is different from the vaccinating centre, appropriate and timely communication of the adverse event should occur.

3 The date and/or time of first observation of the first sign or symptom leading to Neonatal death can be used if date/time of neonatal death is not known.

4 The date and/or time of neonatal death is defined as the date and time post maternal immunization and subsequent birth of the neonate, when the Neonatal death occurred. This may only be possible to determine in retrospect. In cases where date of death is not available, date of issuance of death certificate, date of reporting the event (from source to recipient), and date of burial should be used, whichever is closer to date of death.

5 An AEFI is defined as serious by international standards if it meets one or more of the following criteria: (1) it results in death, (2) is life-threatening, (3) it requires inpatient hospitalization or results in prolongation of existing hospitalization, (4) results in persistent or significant disability/incapacity, (5) is a congenital anomaly/birth defect, (6) is a medically important event or reaction.

6 To determine the appropriate category, the user should first establish, whether a reported event meets the criteria for the lowest applicable level of diagnostic certainty, e.g. Level three. If the lowest applicable level of diagnostic certainty of the definition is met, and there is evidence that the criteria of the next higher level of diagnostic certainty are met, the event should be classified in the higher level of certainty category. This approach should be continued until the highest level of diagnostic certainty for a given event could be determined. Major criteria can be used to satisfy the requirement of minor criteria. If the lowest level of the case definition is not met, it should be ruled out that any of the higher levels of diagnostic certainty are met and the event should be classified in additional categories four or five.

7 If the evidence available for an event is insufficient because information is missing, such an event should be categorised as “Reported Neonatal death with insufficient evidence to meet the case definition”.

8 An event does not meet the case definition if investigation reveals a negative finding of a necessary criterion (necessary condition) for diagnosis (e.g. not a live birth). Such an event should be rejected and classified as “Not a case of Neonatal death”.

9 Use of this document should preferably be referenced by referring to the respective link on the Brighton Collaboration website (http://www.brightoncollaboration.org).
