# Supplementary material for: Neonatal death: Case definition & guidelines for data collection, analysis, and presentation of immunization safety data
Source: Vaccine. 2016 Dec 1;34(49):6027–37. doi: 10.1016/j.vaccine.2016.03.040 (PMC5139812; doi:10.1016/j.vaccine.2016.03.040)
Supplement: Supplementary file 1 [file mmc1.docx]

Rebuttal - Manuscript title: Neonatal death: Case Definition & Guidelines for Data Collection, Analysis, and Presentation of Immunization Safety Data

Date: 31^st^ December 2015

|  | **Review comments** | | **Author reply** |
| --- | --- | --- | --- |
| **1.a. Do you agree with the rationale of the case definition?** | Yes | 12 |  |
|  | No | 0 |  |
|  | Do not know | 1 |  |
| 1.b. If no / do not know, explain why. | In general I understand the rationale of the case definitions, but there are some loose ends. The term "neonatal", it is agreed , refers to the interval after expulsion of a viable fetus up to the completion of day 28 after expulsion.  1.What is not clear is where products of conception dying in utero at any point up to the time of expulsion (or Caesarian section) belong. Clearly, they do not fall in the term "neonatal". | | Foetus which dies prior to birth (expulsion) is considered a stillbirth. A separate GAIA definition document on stillbirths is nearing completion. |
|  | 2.The term "abortion" is commonly used to refer to an induced termination of pregnancy. The situation is not referred to in the definition, and is an event not really covered by the term "neonatal". | | ‚Abortion‘ is a termination of pregnancy prior to foetal viability, but can include spontaneous and induced terminations. |
|  | 3.The terms "abortion" and "miscarriage" should not be confused with neonatal death, as in both cases the death occurs before birth. | | In many instances, an aborted foetus (foetus born before it is considered ‚viable‘) may have signs of life |
|  | 4.It is not clear why vaccination could not be administered before pregnancy to women of childbearing age intending to become pregnant, thereby eliminating part of the problem of AEFI during pregnancy. | | This is a whole topic on its own!! GAIA executive committee has requested to restrict vaccination to DURING pregnancy only. |
|  | 5.While it is true that reporting criteria vary between countries, the stated goal should be to bring them all to an acceptable level of completeness and comparability. | | Agree, but within reason.  It is impossible to impose the definition of a viable newborn on all settings, due to vast differences in accessibility to care |
|  | 6.It is not discussed that, due to differences in degrees of litiginousness between countries, those with high lawsuit rates for medical reasons tend not to require vaccination during pregnancy due to the risk of opportunistic lawsuits in the case of adverse outcomes of pregnancy, despite benefits to public health. | | Vaccination in pregnancy is on the increase in countries with high rates of litiginousness |
|  | 7. There is ambiguity in the text and the recommendations which can confuse readers regarding what is day one of life. Day one should be defined as the first 24 hour period beginning with the time of live birth. Calling this "day zero" will lead to much confusion and a high likelihood that many reporters will ignore this a report events as occurring on day 1, 2, 3..etc. without clarifying whether they are using or ignoring "day zero." The text confusingly refers to "the second day" as "day 1". | | Should be standardised across all GAIA manuscripts. We have addressed this in the manuscript by removing the use of day zero in the manuscript. Instead, 24 hours or 1st day of life has been used and further clarified how to calculate age at death. |
|  | 8.There should be greater clarify about the definition of "live born". If it breathes and moves, and optionally also makes sounds, it should be considered "live born" regardless of viability in a given locale. | | The WHO definition gives examples of signs of life but these signs of life are not restricted to this list only. Sounds emitted by a newborn could be identified as sign of life and included in definition. We have, however, not added it to the WHO definition accepted for this manuscript |
|  | 9.Sections of text are highlighted in dark gray on the  black and white printouts, but what this means is not explained. "Etiology and risk factors" is one such section, and seems inappropriate here because in the birth situation there is not going to be any information on etiology or risk factors in most cases. Similarly, the highlighted items "early birth risk factors" and "late birth infection-related" are left hanging, | | This was a section which was pending upon completion of the literature review which was not included. All sections have now been updated. |
|  | In general the manuscript has so many such issues that it truly does not appear ready for professional peer review. Normally such reviews are carried out on final drafts deemed ready to send to journals, or received by journals for review. This one does not fit the bill. birth | | Acknowledge that the draft manuscript was not a final draft. |
|  |  | |  |
| 1.c. Comments? | 1.Several abbreviations are nowhere translated in the text. This needs careful correction.(eg. NND, LMIC,GA, HIC). | | Done |
|  | 2.There are a number of incomplete entries, e.g. on the title page: 9(Niranjan); "11xxxAmbujam", 14xxx(Alexandra). | | Completed |
|  | 3.I could not find an asterisk next to the Corresponding author's name. | | Flor to finalise |
|  | http://www.brightoncollaboration.org/internet/en/index/working_groups.html does not function.  Just checking to see which names were included. | | Brighton to confirm that website accessible prior to publication. Table of reviewed papers is included in appendix. |
|  | page 4 - incomplete with 000's and yellow highlights. ( I did not see the inventory of case definitions of neonatal death - so the statement can only be true if you send me the inventory now - or alter the statement to state that it was made available to a few of the working group members. | | Now updated |
|  | well written and helpful for all economic strata Countries Data collection globally will be less confusing . | | Thank you |
|  | none | |  |
|  | none | |  |
|  | The Dollfus classification system can be adapted for use with ICD-10 codes to create a comprehensive, etiology-based profile of infant deaths (REF: Nakamura AM et al., Public Health Rep. 2015 Nov-Dec;130(6):632-42). | | We reviewed the Dollfus classification which classifies all possible causes of NND and the relationship to NND.  Our proposal is simpler and designed for a specific purpose for neonatal death following maternal immunization. The Dollfus classification are complementary to this manuscript |
|  | 1. Data about the pregnancy immunization? Based in literature or evidence need to clarify with data the neonatal deaths from immunization and which vaccine  2.I do not think is appropriate to use abbreviation in definition: World Health organization and ICD-10…..(explain ICD-10)  3. 1.3.3 (i) date/time, name of vaccines of immunization of the mother …….. | | 1.  Our aim is not to define causality between neonatal death and maternal vaccinations but rather to correctly classify death according to gestational age at birth and time of death of the baby  2. Amended abbreviations  3. Not clear what the reviewer has requested here |
| **2.a. Does the preamble clearly describe the intended use of the definition?** | Yes | 8 |  |
|  | No | 1 |  |
|  | Do not know | 1 |  |
| 2.b. If no / do not know, explain why. | 1.Numerical units should be conformed, not presented in differing ways, eg. "<1 million" vs "0.8 million". | | Amended |
|  | 2.References could be shown as "[4-5]" rather than [4], [5], and [7-9] rather than [7]-[9]. | | Amended |
|  | 3.The issue of foetal death has been overlooked and will need a separate definition of its own. | | The stillbirth GAIA manuscript covers foetal death |
| 2.c. Comments? | Need to define; NND, LMIC and HIC. | | Amended |
|  | 1.Indicate whether the literature search was confined to English language articles or not. | | Amended |
|  | 2.Delete comman between "maternal immunization" and "maternal vaccination" and substitute "and" in 1.2, para.2. | | Amended |
|  | 3.Delete comma after "maternal vaccination" Ibid | | Amended |
|  | 4.Indicate number or articles reviewed, now appearing as "000" in 1.2 para.2 and also para.3. | | Amended |
|  | 5.Delete ")" after "at all" in 1.2 para.3. | |  |
|  | 6.Add comma in 1.3.2, between "it" and "such". | |  |
|  | 7. In 1.3.3.(which confusingly is Followed by 1.3.2 again) line 3, change "from" to "after" the first day. | | Amended |
|  | 8.In the second section also marked "1.3.3." on Timing of maternal immunization, para 2 last line should read "groupS". | | Amended |
|  | 9.At the same point in text, reference numbers are  needed for Flor Munoz et al and Jim Buttery et al 2015. | |  |
|  | 10.Same section, 2nd para. from page bottom: as noted above, first day should NOT be called "day zero", second "day 1" and third "day 2". Infants born live and still alive 24 hours after birth should be said to have completed their first day of life. | | Addressed through earlier comment by a reviewer. For consistency across all GAIA manuscripts this acceptable definition has been used |
|  | 11.In my view, accepting misdiagnosis and misclassification by any commonly used misnomers will only cloud the issues and add to confusion. Viability should be by clear definition, not vary with country at a given and changing stage of its development.(RE:section on "related terms of neonatal death". | | This manuscript should be applicable to all settings with varying resources. Imposing a specific, inflexible viability will alienate low resourced settings which have to use higher gestational ages to classify viability due to lack of capacity to nurse extremely preterm newborns. |
|  | 12.To reiterate, under shaded section on "Very early", need to note reporter, ideally the pregnant woman herself, giving info on antenatal care or lack thereof. Asking for risk factor info here, and for "infection-related" data seem Quixotic. | | This section has been removed from the manuscript as it has been discussed within different sections of the manuscript. Reporting of events has been addressed in the data collection section |
|  | 13.Item 1.4. It should be stated that if the setting is that of clinical trial, the standards of conduct and information gathering and recording should be the same globally, as ensured by the trial sponsors. Otherwise such a "clinical trial" would amount to garbage and also be unethical. | | Agreed. Sponsors need to be sensitive to the capacity of clinical sites in low resource settings to esure there is consistency of data collection between sites where the capacity of the lowest resourced setting dictates the data collection in other sites. Sites will only be selected by sponsors where there is basic accepted level of competence.This is not possible where clinical trials are conducted in hospitals with limited resources utillising the standard of care available in that setting. Sponsors will create an imbalance where mothers not included in the trial are disadvantaged which is against ethical principles. |
|  | Can neonates donate their organs and/or bone marrow after death? Can this be included in the preamble page 4/5. | | This topic is beyond the scope of this manuscript. |
|  | page 5 1.3.3 - this is still not established. I have not seen any of this material. | | Unclear what the reviewer is referring to |
|  | page 6 - shouldn't maternal infection/sepsis/fever be included as a risk factor? | | Yes. Risk factors are to be collected as stipulated in the data collection section |
|  | page 8 - live born definition not finished. NND not defined | | Done |
|  | page 9 - gestational age does not have a definition -- by fundal height measurement? by LMP? by ultrasound? | | Gestational age assessment should be based on Levels of certainty for gestational age being developed by the preterm delivery GAIA working group (Buttery et al) |
|  | page 9 -- why do you need to have a medically confirmed death in any of the definitions? isn't death a vital statistic collected in all countries? | | In many settings, confirmation of death by a nationally accepted, medically qualified individual is required to issue a death certificate. Vital statistics are often based on the review of registered deaths by local or national government authorities in different settings and are responsible for reporting death statistics. |
|  | very clear outline to include all possible diagnostic criteria | |  |
|  | none | |  |
|  | none | |  |
|  | The preamble captures very well the differences in society's socioeconomic capacity to recognize as well as record a neonatal death. | |  |
| **2(2).a. Do you agree with the definition?** | Yes | 3 |  |
|  | No | 0 |  |
|  | Do not know | 0 |  |
| 2(2).b. If no / do not know, explain why. |  | |  |
| 2(2).c. Comments? |  | |  |
| **3.a. Do you agree with Level 1 of diagnostic certainty?** | Yes | 7 |  |
|  | No | 2 |  |
|  | Do not know | 1 |  |
| 3.b. If no / do not know, explain why. | Generally I agree but there are caveats:  1.Under item 2, the footnote 1 after "terms for use below" does not seem to relate to the footnote 1 at the bottom of the page. Explain. | | Footnote removed and terms utilized in levels of certainty have been defined earlier in the section. |
|  | 2.In para.2, need a reference for "Buttery..." | | Agree. This is a document in development, and will be released simultaneously with this manuscript. Reference will be updated. |
|  | 3.Item "1" superscript after "2", says "per definition to be finalized in manuscript (Neonatal death). " What does this mean?? I thought this WAS the manuscript. | | Amended |
|  | 4.The top left box in the dendrogram is double printed over and unreadable. | | Amended |
|  | 5.In 2.1, "miscarriage" and "abortion" are NOT neonatal | | Amended |
|  | 6.Under 2.1, level 3, need to state how the parent/family member/ attendant would possibly be able to know the gestational age. Also, does "parent" mean "the pregnant woman or mother"? Or her parent? | | Level 3 will be used when no other accurate measure of GA I available. Approximate GA would be on history of mother, from her knowledge of her own menstrual cycles. In LMIC many women are acutely aware of their cycles. |
|  |  | |  |
|  | There is no reason for medically confirmed death to be a level 1 criterion. page 11 - yes | | We’ve already addressed in earlier comment |
|  | Live birth infant has not been clearly defined especially for a preterm and non-viable infant | | We have indicated that the live birth definition applies to births at any gestational age |
| 3.c. Comments? | You reversed the ">" sign giving impression of less than 22weeks instead of greater than 22 weeks.  Same error for weight of 500g | | Non-viable definition is classified as „<“ (less than) 22 weeks whereas viable definitions are ≥ (greater than or equal to) 22weeks |
| **4.a. Do you agree with Level 2 of diagnostic certainty?** | Yes | 10 |  |
|  | No | 0 |  |
|  | Do not know | 0 |  |
| 4.b. If no / do not know, explain why. |  | |  |
| 4.c. Comments? | What is the difference between a "community Member" or any other lay witness? | | This list is an example of a reporter. A lay witness is acceptable (line 565/96) |
|  | above about who determines death | |  |
|  | You reversed the ">" sign giving impression of less than 22weeks instead of greater than 22 weeks.  Same error for weight of 500g | | Non-viable definition is classified as „<“ (less than) 22 weeks whereas viable definitions are ≥ (greater than or equal to) 22 weeks |
|  | except as mentioned above | |  |
| **5.a. Do you agree with Level 3 of diagnostic certainty?** | Yes | 9 |  |
|  | No | 1 |  |
|  | Do not know | 0 |  |
| 5.b. If no / do not know, explain why. | Apart from reversal of the ">" sign, I would think that it should be greater than 6 months not 4 months in developing countries | | Non-viable definition is classified as „<“ (less than) 22 weeks whereas viable definitions are ≥ (greater than or equal to) 22weeks.  Amended non viable period to 5 months which equates to greater than 20 weeks which is more in line with our 22 week cut-off of non-viability |
| 5.c. Comments? | except as mentioned in level 1 | |  |
| **6.a. Do you agree with the footnotes of the case definition?** | Yes | 8 |  |
|  | No | 0 |  |
|  | Do not know | 1 |  |
| 6.b. If no / do not know, explain why. |  | |  |
| 6.c. Comments? | Make sure to define abbreviations used. | | Amended |
|  | clinical and immunization history - page 12 -- is this self report or documented by record?, esp food allergies, and allergies to vaccine components.  page 13 - 3.1.4 - patient could be seen by NP or PA instead of a physician as listed -- physician should be replaced by generalist, ped, FM, ob, pathologist, NP, PA and others from the first document. | | Added statement on reporting by either review of medical charts, confirmation by any health care provider or history as reported by patient ( line 709) |
|  | It still seems incomplete at various places | | Now completed |
| **7.a. Is the definition applicable in your setting?** | Yes | 8 |  |
|  | No | 0 |  |
|  | Do not know | 2 |  |
| 7.b. If no / do not know, explain why. | See earlier comments on issue of "setting". | | Addressed in earlier comment |
| 7.c. Comments? | Variable levels of health care in urban and rural areas of India may result in variable data | | Agree. Variability in access to facilities has led to development of levels of certainty with level 3 being the minimally acceptable report of data to define a neonatal death. |
| **8.a. Do you agree with the guidelines?** | Yes | 12 |  |
|  | No | 0 |  |
|  | Do not know | 0 |  |
| 8.b. If no / do not know, explain why. |  | |  |
| 8.c. Comments? | There is a question at end of Doc that asks for "treatment given" but this is vague and not defined. | | Addressed in line 758 |
|  | 1.Replace highlighted term "numbers" under 3.1 at page bottom. | | Done |
|  | page 18 - notes for guidelines - note 8: specify that level 3 is lower than level 1. | | See comment in section 3.1.6 (line 403) |
|  | 2.Just before 3.1.1., Explain what "data" on maternal participant is meant here. | | Clarified in line 635 |
|  | 3.3.1.2., item 6; indicate date of birth of vaccinee or of infant. | | Amended |
|  | 4. 3.1.2., item 8, indicate what is intended by the vague term "identification of indicators". Also, add "i.e." after common on line 3, preceding "a history of asthma". | | Omitted ‘identification of’ from manuscript. |
|  | 5.Ibid, item 9: would suggest adding "time of last noted signs of fetal life and indicate what they were". | | All infants fulfilling neonatal death classification are live births and this comment is applicable to stillbirth definition. |
|  | 6.3.1.3., item 14: add "nature and quantity of diluent used". | | amended |
|  | 7.Ibid, item 16: State what other injection devices there might be (not clear whether there are any others). | | Amended |
|  | 8.Ibid, item 19. State clearly whether "patient" refers to the pregnant woman or the fetus/neonate | | Amended. Patient refers to neonate |
|  | 9.Ibid, item 22: state if this is intended to mean "measured maternal parameters". | | Amended to neonate parameters |
|  | 10.3.1.4. Section 25: The business about "seriousness of event, etc." seems redundant, since by definition , death, neonatal or other , is "serious". | | Agreed and removed |
|  | 11.Ibid, item 25: add "any medications" before "food." | | Done |
|  | 12.3.1.5. This section makes no sense, because the duration of surveillance for neonatal death is defined by the duration of the birth process, not by biologic or other characteristics of any vaccine or disease, etc. | | This section is necessary as follow up of the neonate for adverse events can only be conducted for specified risk periods and not necessarily for the entire neonatal period. |
|  | 13.Ibid, item 28, line 2: add semicolon after "predefined", and comman after "likewise; small case for "it" and delete "aim to" | | We have deleted this statement |
|  | 14.Unclear what is meant by "if this is not feasible" in  3.1.5. item 32, line 3. How could it not be feasible? | | By feasible we mean limitations in study design and resources which does not allow follow up neonates throughout the study period (line 812) |
|  |  | |  |
|  |  | |  |
|  |  | |  |
|  | But the table mentioned in page 15/22 needs to be made more detailed. Interval of immunization and birth should be stratified further- >30 days should be split to 30-60 days.60-90 days and more as most vaccinations of preganancy are given earlier and need to be observed for trends in neonatal death | |  |
| **9. Any general comments?** | I would consider asking the mother experiencing a neonatal death an opened-ended question of why she thought she miscarriage. | | Unclear if reviewer means miscarriage or neonatal death. In any case, |
|  | Add the specific names to the acknowledgements section. | | To follow (Flor, please assist) |
|  | Except for my objection above, okay. | |  |
|  | very useful to all of the counties with variable income status and data collection is better. identifying the cause of death as best as possible will help immunization practices and much needed advantages to improve preventable deaths | |  |
|  | Some categories of sudden sleep-related neonatal/infant deaths may overlap within Level 3 of diagnostic certainty. | | If a sudden infant deaths falls within the neonatal period, then yes, there will be some overlap |
|  | The guidelines seem well intentioned but are still incomplete. there is not enough evidence to look through and the trends for immunization-birth-neonatal death need to be observed over a longer time period to make conclusions as an AEFI | | This case definition and guideline is intended to contribute to monitoring of trends in maternal immunization and neonatal death in a standardized manner. We do not attempt to provide insight into whether neonatal death is an adverse event following maternal immunization |
| **7. Any other comments you would like to share?** | A very well written manuscript. Please harmonize the description of days at deaths. Neonatal deaths have been described as 0-7 completed days, while 0 completed days doesn't mean anything. Either write 0-6 days of life or 1-7 completed days. | | Addressed in earlier comments |
| **3.a. Based on your study setting and the procedures / diagnostic requirements for the different levels of certainty in the case definition, would you be able to obtain all levels of certainty?** | Yes | 3 |  |
|  | No | 0 |  |
|  | Do not know | 0 |  |
| 3.b. If no / do not know, explain which level(s) would not be applicable and why (e.g. not having all procedures available in countries where study is done / retrospective assessment of charts would not allow information / exclusion / negative criteria to be found consistently) |  | |  |
| 3.c. Comments? | I think is good work and help so much healthcare workers. | |  |
